# Concordance of Chronotype Categorisations Based on Dim Light Melatonin Onset, the Morningness-Eveningness Questionnaire, and the Munich Chronotype Questionnaire

**DOI:** 10.3390/clockssleep3020021

**Published:** 2021-06-17

**Authors:** Andrew M. Reiter, Charli Sargent, Gregory D. Roach

**Affiliations:** Appleton Institute for Behavioural Science, Central Queensland University, Goodwood, SA 5034, Australia; charli.sargent@cqu.edu.au (C.S.); greg.roach@cqu.edu.au (G.D.R.)

**Keywords:** chronotype, concordance, DLMO, MEQ, MCTQ, categorisation, cut-off, early, intermediate, late

## Abstract

Chronotype reflects circadian timing and can be determined from biological markers (e.g., dim light melatonin onset; DLMO), or questionnaires (e.g., Morningness-Eveningness Questionnaire; MEQ, or Munich Chronotype Questionnaire; MCTQ). The study’s aim was to quantify concordance between chronotype categorisations based on these measures. A total of 72 (36f) young, healthy adults completed the MEQ and MCTQ and provided saliva samples hourly in dim light during the evening in a laboratory. The corrected midpoint of sleep on free days (MSF_sc_) was derived from MCTQ, and tertile splits were used to define early, intermediate and late DLMO-CT, MEQ-CT and MSF_sc_-CT chronotype categories. DLMO correlated with MEQ score (*r* = −0.25, *p* = 0.035) and MSF_sc_ (*r* = 0.32, *p* = 0.015). For early, intermediate and late DLMO-CT categories, mean(SD) DLMO were 20:25(0:46), 21:33(0:10) and 23:03(0:53). For early, intermediate and late MEQ-CT categories, mean(SD) MEQ scores were 60.5(5.3), 51.4(2.9) and 40.8 (5.0). For early, intermediate and late MSF_sc_-CT categories, mean(SD) MSF_sc_ were 03:23(0:34), 04:37(0:12) and 05:55(0:48). Low concordance of categorisations between DLMO-CT and MEQ-CT (37%), and between DLMO-CT and MSF_sc_-CT (37%), suggests chronotype categorisations depend on the measure used. To enable valid comparisons with previous results and reduce the likelihood of misleading conclusions, researchers should select measures and statistical techniques appropriate to the construct of interest and research question.

## 1. Introduction

The body clock of each human individual is uniquely timed. Individual differences in body clock timing impact biological rhythms, resulting in a wide range of preferred and actual daily sleep and activity patterns. Chronotype is a construct that reflects individual differences underlying circadian timing [1,2]. Earlier chronotypes get up, achieve peak performance, and go to bed earlier than later chronotypes [3]. Mounting evidence suggests chronotype plays an important role in physical health (e.g., diabetes, metabolic disorders), mental health (e.g., depression, anxiety), and shift-work tolerance [4,5]. 

Chronobiology researchers have applied varied approaches to the operationalisation and analysis of chronotype for more than one hundred years. Chronotype operationalised as circadian phase or timing can be based on the timing of objective markers in biological variables that exhibit circadian rhythms (e.g., melatonin concentration, core body temperature). Dim light melatonin onset (DLMO) is the most common and reliable circadian phase marker and can be detected in periodic blood, saliva and urine samples [6]. However, collection of biological samples is invasive, laborious, and costly, limiting chronotype determination from objective measures to laboratory experiments with small samples [3]. Self-report questionnaires are simple to administer, and several have been developed to determine circadian preference or typical timing. Chronotype operationalised as preferred timing of sleep and activity is usually determined from the Morningness-Eveningness Questionnaire (MEQ) [7]. Chronotype operationalised as the phase of entrainment between the sleep-wake cycle and the 24-h clock can be based on sleep markers sourced from the Munich Chronotype Questionnaire (MCTQ) [8], which incorporates questions about typical times for sleep and activity. 

Inconsistency in the approach to the operationalisation of chronotype has stimulated discussion in the literature [1,9]. Whilst chronotype based on objectively-measured biological rhythms is considered appropriate for examination of the impact of circadian timing on outcome variables (e.g., cognitive performance) [10], chronotype based on daily preference is appropriate for research on psychological traits, and chronotype based on the phase of entrainment is appropriate for research on circadian traits that are influenced by the entraining environment [9]. Several studies have assessed relationships between biological rhythms and MEQ/MCTQ measures, e.g., [11,12,13]. DLMO consistently correlates with both MEQ score and MSF_sc_ (corrected midpoint of sleep on free days derived from the MCTQ), however the strength of correlations varies between populations (e.g., DLMO-MEQ score: *r* = −0.40, DLMO-MSF_sc_: *r* = 0.54 [14]; DLMO-MEQ score: *r* = −0.70, DLMO-MSF_sc_: *r* = 0.68 [2]). Although measures of daily preferences and phase of entrainment may correlate, they are not comparable or interchangeable [9]. It follows that any research applying circadian typology should clearly justify the selection of the chronotype definition and measure. Furthermore, to simplify analyses and interpretations, distributions of continuous measures are often stratified into chronotype (e.g., early, intermediate, and late) categories. However, the approach to determining the cut-offs that separate categories is also inconsistent; most researchers apply percentile splits, e.g., [15,16], however, others use self-decided or recommended absolute cut-offs, e.g., [17,18].

Although DLMO, MEQ score and MSF_sc_ correlate, chronotype categorisations based on them do not necessarily concord. An individual may be categorised as early-type based on one measure, but as intermediate or late-type based on another measure. Low concordance between categorisations based on different chronotype measures and arbitrary cut-off scores limit meaningful comparisons of results between studies [1]. Some researchers have sought to mitigate this issue by categorising using a combination of subjective and objective measures, e.g., [19]. A weighted combination of multiple continuous circadian measures may provide more reliable chronotype categorisations, and several approaches have been suggested [20,21].

Surprisingly, there do not appear to be any studies that have systematically quantified concordance between chronotype classifications based on independent, subjective and objective circadian measures [22]. This study will close this gap by quantifying the concordance of chronotype categorisations based on an objective measure (DLMO) with categorisations based on two subjective measures (MEQ and MCTQ), for a relatively large sample of participants. As MSF_sc_ (from MCTQ) is a measure of behaviour influenced by circadian timing, we predict greater concordance between DLMO and MCTQ chronotype categorisations than between DLMO and MEQ chronotype categorisations.

## 2. Results

### 2.1. DLMO, MEQ Score and MSF_sc_

**Figure 1:**
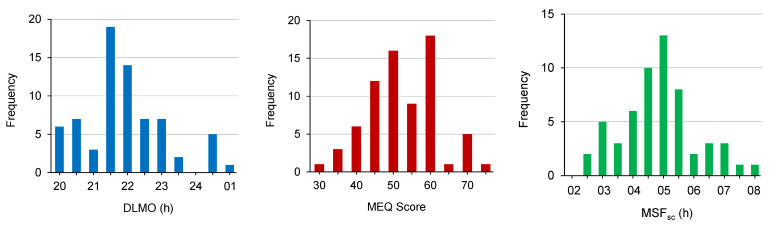
Frequency distributions of DLMO (*n* = 71), MEQ score (*n* = 72), and MSF_sc_ (*n* = 57).

**Figure 2:**
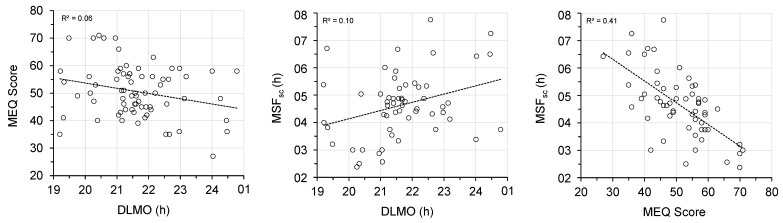
Scatter plots of MEQ score versus DLMO (*n* = 71), MSF_sc_ versus DLMO (*n* = 57), and MSF_sc_ versus MEQ score (*n* = 57).

The MEQ was completed by each of the 72 participants, and DLMO was available for 71 participants. MSF_sc_ was calculated for the 57 participants who reported not using an alarm to wake up on free days on the MCTQ. Of these 57 participants, 28 (i.e., 49%) reported they had regular work schedules. Kolmogorov–Smirnov tests confirmed the DLMO, MEQ score, and MSF_sc_ distributions were normal; *D* (71) = 0.10, *p* = 0.076; *D* (72) = 0.066, *p* = 0.200; *D* (57) = 0.099, *p* = 0.200, respectively (Figure 1). Pearson bivariate correlation analyses confirmed DLMO and MEQ score were correlated, *r* (71) = −0.25, *p* = 0.035, DLMO and MSF_sc_ were correlated, *r* (57) = 0.32, *p* = 0.015, and MEQ score and MSF_sc_ were correlated, *r* (57) = −0.64, *p* < 0.001 (Figure 2).

### 2.2. DLMO-CT Categories 

DLMO and MEQ data were available for the 57 participants for which MSF_sc_ could be calculated. The DLMO distribution for this sub-sample was divided into DLMO-CT categories; participants with DLMO earlier than 21:15 were categorised as early DLMO-CT, participants with DLMO later than 21:57 were categorised as late DLMO-CT, and the remaining participants were categorised as intermediate DLMO-CT. The corresponding mean MEQ scores and mean MSF_sc_ were calculated for each DLMO-CT category (Table 1). A one-way between-groups ANOVA with DLMO-CT as the independent variable revealed no significant effect for MEQ score, *F*(2,54) = 2.12, *p* = 0.13, and a significant effect for MSF_sc_, *F*(2,54) = 4.79, *p* = 0.012. Post-hoc comparisons using the Games–Howell procedure revealed a difference between the mean MSF_sc_ of the early and late categories (*p* = 0.027), but no differences between mean MSF_sc_ of the other categories: early-intermediate (*p* = 0.083) and intermediate-late (*p* = 0.585).

### 2.3. MEQ-CT and MSFsc-CT Categories 

For the sub-sample of 57 participants, the MEQ and MSF_sc_ distributions were each divided into chronotype categories. For the MEQ score distribution, participants with scores greater than or equal to 56 were categorised as early MEQ-CT, participants with scores less than or equal to 46 were categorised as late MEQ-CT, and the remaining participants were categorised as intermediate MEQ-CT (Table 2a). For the MSF_sc_ distribution, participants with MSF_sc_ earlier than 04:16 h were categorised as early MSF_sc_-CT, participants with MSF_sc_ later than 04:53 h were categorised as late MSF_sc_-CT, and the remaining participants were categorised as intermediate MSF_sc_-CT (Table 2b).

### 2.4. Concordance of Categorisations

To assess the concordance of categorisations based on the three measures, DLMO-CT categorisations were compared with MEQ-CT and MSF_sc_-CT categorisations for each participant. The number of participants for each of the nine permutations was tallied (Table 3, Part A). The number of concordances was calculated as the number of participants whose DLMO-CT categorisation matched the subjective measure categorisation (i.e., early-early, intermediate-intermediate, late-late). The number of 1-step errors was calculated as the number of participants for which there was a one-category difference between the DLMO-CT category and the subjective measure category (i.e., early-intermediate, intermediate-early, intermediate-late, late-intermediate). The number of 2-step errors was calculated as the number of participants for which there was a two-category difference between the DLMO-CT category and the subjective measure category (i.e., early-late, late-early) (Table 3, Part B).

## 3. Discussion

For this sample of young, healthy adults, the continuous measures of DLMO, MEQ score and MSF_sc_ were normally distributed and correlated. Circadian questionnaires such as the MEQ and MCTQ are simple and suited to the field and large-scale studies, but their measures are not interchangeable with each other, or with DLMO. DLMO was more strongly correlated with MSF_sc_ than MEQ score, although neither correlation is strong. MSF_sc_, a subjective measure of sleep timing, appears more closely related to endogenous circadian timing than the subjective preference for sleep timing measured by MEQ [2]. The correlation between DLMO and MEQ score (*r* = −0.25) is weaker than the same correlation reported for other studies (*r* = −0.35 to −0.76) [23]. The correlation between DLMO and MSF_sc_ (*r* = 0.32) may be weaker than the same correlation reported by others (*r* = 0.54 [14], *r* = 0.68 [2]) because our participants were mostly international travellers or students. Application of the MCTQ to individuals with irregular work arrangements may be problematic, as its calculations assume structured work schedules [24]. Furthermore, chronotype can only be determined from the MCTQ when an alarm clock is not used on free days [25], and therefore MEQ is sometimes used in preference to MCTQ simply to maximise available data, e.g., [26]. In the present study, MSF_sc_ could not be calculated for 14 participants, reducing our sample to 57. A recently developed short-form of MCTQ (µMCTQ) allows MSF_sc_ to be calculated for all respondents by capturing wake times only on free days on which an alarm clock is not used [27], and a similar modification to the standard MCTQ may enhance its utility.

For chronotype categorisations based on DLMO, there were no differences in mean MEQ scores between each category, and only a difference in mean MSF_sc_ between the early and late categories. This suggests that for this sample, MEQ scores and MSF_sc_ of participants in the early, intermediate, and late chronotype categories based on DLMO were indistinguishable. Chronotype categorisations based on splits of both the MEQ score distribution and the MSF_sc_ distribution had low concordance with chronotype categorisations based on a split of the DLMO distribution. Compared with categorisations based on DLMO, concordance for categorisations based on both MEQ score and MSF_sc_ was 37% (i.e., 63% error rate). However, categorisations based on the MEQ score had a greater proportion of 2-step errors (21%) than categorisations based on MSF_sc_ (14%). The low concordance may be a consequence of applying percentile splits to normally distributed continuous variables to create a categorical variable. This approach allows for the use of ANOVA statistical methods that simplify analyses and interpretation of differences between groups. A shortcoming of this methodology is that participants with measures near, but below or above, the percentile cut-offs can be assigned to different categories, although their measures are actually closer to each other than other members of their assigned category. Secondly, percentile splits of each study sample will yield different thresholds, limiting the generalisability of the results and comparisons with other studies. Although samples are sometimes dichotomised by a median split, e.g., [26], trichotomisation by tertile split, as in the present study, should provide superior between-group comparisons. The risk of drawing incorrect conclusions arising from categorisations based on continuous measures can usually be mitigated or overcome by regression and correlational analysis [28].

An important limitation of this study is that extreme chronotypes were excluded due to the selection criteria for the main shift work-study. However, our findings are generalisable to populations of younger, healthy adults, as epidemiological studies report that extreme chronotypes are uncommon [29]. Mean DLMO of our early and late DLMO chronotype categories (20:25 h and 23:03 h respectively) are comparable with criteria applied in other studies (e.g., early: <21:30 h, late >22.30 h [20]), indicating our sample included a broad range of chronotypes. We demonstrated greater concordance between categorisations based on DLMO and categorisations based on each of the subjective measures for the early and late categories than for the intermediate category, suggesting concordance may be greater with more extreme chronotypes. A second limitation to our study is that to illustrate the underlying measurement issue, we divided our sample into chronotype categories using tertile splits. Although this method is commonly used by chronotype researchers, categorisations based on other arbitrary or recommended percentiles or absolute cut-offs would likely yield different levels of categorisation concordance.

Valid measurement is a key element of scientific research. Our results suggest that laboratory experimental research on the differential effects of body clock timing on outcome variables should use objective biological measures where possible. DLMO, MEQ and MCTQ measure related, but different constructs that are not interchangeable. Although these measures were correlated for this sample, the correlations were not strong. Consequently, chronotype categorisations based on percentile splits of these distributions showed low concordance. To enable valid comparisons with previous results and reduce the likelihood of misleading conclusions, chronotype researchers should select the most appropriate measures and statistical techniques for their construct of interest and research question.

## 4. Materials and Methods

### 4.1. Participants

Data were collected during a simulated shift work-study conducted at the Appleton Institute in Adelaide, South Australia. Participants were 72 young, healthy adults (36 females, 36 males) with a mean (± *SD*) age of 23.1 (± 3.6) years and body mass index (BMI) of 21.5 (±1.9) kg/m^2^, recruited by advertisements posted at hostels, student accommodation and university campuses and on casual employment websites. Screening involved the completion of a general health questionnaire and an in-person interview. Key inclusion criteria included age (18–30 years), BMI (18–25 kg/m^2^) and good physical and mental health. Key exclusion criteria included smoking, use of medications (excluding oral contraceptives), use of recreational drugs, excessive alcohol or caffeine consumption, excessive exercise and shift work or transmeridian travel during the month prior to the study. Participants were mostly international travellers or students, provided written informed consent and were financially compensated with an honorarium.

### 4.2. Procedure

Data for this study were collected during the first two days of a multi-day laboratory shift work-study. During the week before the study, participants were requested to maintain their normal sleep patterns, complete a sleep diary, and wear an activity monitor on their non-dominant wrist. In the laboratory, each participant was accommodated with their own bedroom and bathroom. Days 1 and 2 were work-free days. On Day 1, participants entered the laboratory at 16:00 and were provided with a 9-h sleep opportunity (23:00–08:00). On Day 2, participants completed the MEQ and MCTQ and were familiarised with the simulated shift-work tasks between periods of free time. Nine saliva samples were then collected from each participant hourly in dim light (<10 lux) (19:00–03:00). Twenty minutes before each sample was collected, participants were instructed to gently rinse their mouths with water, remain seated, and refrain from eating and drinking until after the sample was collected. To collect saliva, participants rolled a cotton swab in their mouths for approximately 2–3 min. Swabs were refrigerated prior to centrifuging and freezing at −20 °C. After saliva sampling, participants were provided a 9-h sleep opportunity (03:00–12:00).

### 4.3. Measures

#### 4.3.1. Dim Light Melatonin Onset (DLMO)

DLMO was determined from saliva collected using cotton swabs (Salivette; Sarstedt, Nümbrecht, Germany). Melatonin concentration was measured by 4.3 pM direct radioimmunoassay, using reagents from Buhlmann Laboratories AG (Allschwill, Switzerland). DLMO was defined as the time melatonin concentration reached a fixed threshold of 10 pM and stayed above this threshold for at least two subsequent samples [30]. For one participant, whose melatonin concentration was above 10 pM for all samples, a higher relative threshold equal to the mean of the first three melatonin concentration values plus two standard deviations of those values was used [30]. Linear interpolation was applied to estimate the time of DLMO between the sample times immediately before and after concentration exceeded the threshold.

#### 4.3.2. Morningness-Eveningness Questionnaire

The Morningness-Eveningness Questionnaire (MEQ) [7] is the most commonly used circadian typology questionnaire and consists of 19 questions with Likert responses about an individual’s preferred bedtimes, get-up times, and times for activity [22]. Responses to questions are scored and summed to produce an overall morningness score ranging from 16 to 86, with higher scores indicating greater morningness [7].

#### 4.3.3. Munich Chronotype Questionnaire

The Munich Chronotype Questionnaire(MCTQ) [8] incorporates 14 questions about the timing of sleep and activity on work and free days. Responses can be used to assess work schedule, the timing of sleep and alarm clock use on work and free days and the number of days worked per week [31]. The midpoint of sleep on free days (MSF) can be extracted from the MCTQ and used to determine chronotype [8]. The premise of using MSF to determine chronotype is that an individual’s sleep and wake timing on free days corresponds to their circadian timing, with earlier MSF corresponding with earlier chronotype [15]. MSF is calculated as the sleep onset on free days (SO_f_), plus half the sleep duration on free days (SD_f_). As most individuals accumulate a sleep debt during the working week and extend their sleep on free days, MSF can be “sleep-corrected” to MSF_sc_, the corrected sleep midpoint on free days. MSF_sc_ is calculated as MSF minus a correction for sleep debt equal to half the difference between sleep duration on free days (SD_f_) and average sleep duration over the week (SD_week_), which is only applied if sleep duration on free days is greater than sleep duration on workdays [24]. An important limitation of the MCTQ is that individuals who use an alarm to wake up on free days cannot be chronotyped, as their MSF may be impacted and therefore not reflect internal timing [25]. 

### 4.4. Statistical Analyses

Data were analysed using IBM SPSS Statistics for Windows, Version 26.0 (Armonk, NY, USA), with statistical significance, determined using an alpha level of 0.05. Kolmogorov–Smirnov tests were used to assess the normality of the DLMO, MEQ and MSF_sc_ distributions. Pearson pairwise bivariate correlation analyses were used to assess relationships between DLMO, MEQ and MSF_sc_. Consistent with the approach recommended for comparing chronotypes based on mid-sleep times derived from MCTQ within a sample [32], the DLMO, MEQ and MSF_sc_ distributions were each divided into early, intermediate and late DLMO-CT, MEQ-CT, and MSF_sc_-CT categories by applying tertile splits.

## Figures and Tables

**Table 1:** Means, standard deviations, and ranges of DLMO, MEQ Score and MSF_sc_ for early, intermediate and late DLMO-CT categories.

DLMO-CT Category	*n*	DLMO (hh:mm) *M* (*SD*)*, Range*	MEQ Score *M* (*SD*), *Range*	MSF_sc_ (hh:mm) *M* (*SD*), *Range*
Early	19	20:25 (0:46), 19:12–21:13	54.8 (11.1), 35–71	04:01 (1:15), 02:22–06:42
Intermediate	19	21:33 (0:10), 21:16–21:53	49.6 (6.5), 39–60	04:46 (0:47), 03:20–06:40
Late	19	23:03 (0:53), 22:04–00:47	49.2 (10.1), 27–63	05:07 (1:13), 03:22–07:45
Overall	57	21:40 (1:16), 19:12–00:47	51.2 (9.7), 27–71	04:38 (1:11), 02:22–07:45

**Table 2:** (**a**) Means, standard deviations, and ranges of MEQ Score for MEQ-CT categories; (**b**) Means, standard deviations and ranges of MSF_sc_ for MSF_sc_-CT categories.

(a) MEQ-CT		(b) MSF_sc_-CT	
MEQ-CT Category	MEQ Score *n, M* (*SD*), *Range*	MSF_sc_-CT Category	MSF_sc_ (hh:mm) *n*, *M* (*SD*), *Range*
Early	22, 60.5 (5.3), 56–71	Early	19, 03:23 (0:34), 02:22–04:15
Intermediate	15, 51.4 (2.9), 47–55	Intermediate	19, 04:37 (0:12), 04:17–04:52
Late	20, 40.8 (5.0), 27–46	Late	19, 05:55 (0:48), 04:54–07:45
Overall	57, 51.2 (9.7), 27–71	Overall	57, 04:38 (1:11), 02:22–07:45

**Table 3:** **Part A**: Number of participants in each DLMO-CT and MEQ-CT category combination and each DLMO-CT and MSF_sc_-CT category combination. **Part B**: Number (percentage) of concordances, 1-step errors and 2-step errors between DLMO-CT and MEQ-CT categorisations and between DLMO-CT and MSF_sc_-CT categorisations.

**Part A**								
**DLMO-CT Category**	**MEQ-CT Category**				**MSF_sc_-CT Category**			
	**Early**	**Intermediate**	**Late**	**Total**	**Early**	**Intermediate**	**Late**	**Total**
Early	9	5	5	19	8	4	7	19
Intermediate	6	5	8	19	10	5	4	19
Late	7	5	7	19	1	10	8	19
Total	22	15	20	57	19	19	19	57
**Part B**								
Concordance ^1^	9 + 5 + 7 = 21 (37%)				8 + 5 + 8 = 21 (37%)			
1-step errors ^2^	5 + 6 + 8 + 5 = 24 (42%)				4 + 10 + 4 + 10 = 28 (49%)			
2-step errors ^3^	5 + 7 = 12 (21%)				7 + 1 = 8 (14%)			
Total	57 (100%)				57 (100%)			

^1^ Concordance: Agreement between DLMO-CT categorisation and subjective measure categorisation. ^2^ 1-step error: One category difference between DLMO-CT categorisation and subjective measure categorisation (i.e., early or late DLMO-CT categorisation, and intermediate categorisation based on the other measure, and vice versa). ^3^ 2-step error: Two category difference between DLMO-CT category and subjective measure category (i.e., early (late) DLMO-CT categorisation and late (early) categorisation based on the other measure and vice versa).

## Data Availability

The data for this study are not currently publicly available as they are part of a larger dataset that will be used for another purpose.
